# A systematic performance comparison of two Smooth Pursuit detection algorithms in Virtual Reality depending on target number, distance, and movement patterns

**DOI:** 10.16910/jemr.15.3.9

**Published:** 2023-05-29

**Authors:** Sarah-Christin Freytag, Roland Zechner, Michelle Kamps

**Affiliations:** TU Berlin, Berlin, Germany

**Keywords:** Eye movement, Eye Tracking, Smooth Pursuit, VR, Virtual Reality, correlation-based algorithm, vector-angle based algorithm

## Abstract

We compared the performance of two smooth-pursuit-based object selection algorithms in Virtual Reality (VR). To assess the best algorithm for a range of configurations, we systematically varied the number of targets to choose from, their distance, and their movement pattern (linear and circular). Performance was operationalized as the ratio of hits, misses and non-detections. Averaged over all distances, the correlation-based algorithm performed better for circular movement patterns compared to linear ones (F(1,11) = 24.27, p < .001, η² = .29). This was not found for the difference-based algorithm (F(1,11) = 0.98, p = .344, η² = .01). Both algorithms performed better in close distances compared to larger ones (F(1,11) = 190.77, p < .001, η² = .75 correlation-based, and F(1,11) = 148.20, p < .001, η² = .42, difference-based). An interaction effect for distance x movement emerged. After systematically varying the number of targets, these results could be replicated, with a slightly smaller effect.

Based on performance levels, we introduce the concept of an optimal threshold algorithm, suggesting the best detection algorithm for the individual target configuration. Learnings of adding the third dimension to the detection algorithms and the role of distractors are discussed and suggestions for future research added.

## Introduction

The use of the human gaze to interact with machines or software has become a viable alternative to traditional means of input. Compared to mouse control, gaze-based interaction techniques can be faster and particularly useful in situations where both hands are needed to perform a task (29) or in hygiene-critical situations, such as surgery (24).

Especially smooth pursuit movements have proven suitable to provide a range of unobtrusive interaction methods, that allow a broad range of users to interact effectively with gaze-controlled interfaces. Applications range from novel takes on gaze-spelling that let users select their target letter by simply following its’ movement with their eyes (6; 16; 23) to controlling smart-phone applications by observing the movement speed of icons for applications, that, after surpassing a specific matching-criterion, will then be opened (11). The ease of use and usage of very natural gaze movements make these interactions also suitable for interactions in public spaces (15; 33) and have shown promising results when tested with large databases of users (12).

One of the great advantages of employing smooth-pursuit for interaction is the reduction of the Midas touch problem, which states that for interactions that require dwell-time-based approaches, a distinction between a resting gaze that indicates the intention to select and one that was evoked by the wish to examine cannot sufficiently be made (14; 32).

All these applications use one of two algorithms to compare the eye movements of the user with the movement patterns of the UI elements: a correlation-based algorithm, using the Pearson’s product-moment correlation, and an algorithm based on vectors using the Euclidean distance. These algorithms are well-researched for interactions on a 2D-plane. In addition to these, Drewes et al. (9) introduced a novel slope approach, using the slope of a linear regression line for object detection, showing a possible detection for up to 160 individual objects, based on circular movement on several rings of objects. However, this approach was tested in 2D as well.

Since the introduction of the Oculus Rift DK1 at the end of 2012 (18), the technological progress as well as the availability of Head-Mounted Displays (HMDs) for the consumer market have skyrocketed (13). The integration of eye-tracking technology into HMDs followed suit. In only a span of a few years the solutions developed from research editions provided by eye-tracking manufacturers over clip-in solutions to, finally, the mass-production of consumer-level hardware with eye-tracking integrated by default (35). This widespread availability of eye-tracking data during usage of HMDs opens the door for integrating gaze-based interactions by default into consumer media. It also provides researchers with an abundance of opportunities to investigate the transferability of what is known to work in 2D to 3D virtual reality applications.

The natural navigation of the visual space provided by HMDs suggests that the observed gaze behavior would be close to natural, with no artificial affordances of control disrupting the visual exploration of the virtual world Due to this, VR could potentially overcome shortcomings of lab experiments by providing a semi-realistic experience that surpasses artificial lab settings (5; 21). However, there also are challenges unique to experiences of VR via HMDs. One is the users' potential ability to physically move across the 3D environment. Khamis et al. (17) investigated the influence of user movement, target size, the distance to targets, and the radius of circular object trajectories on the performance of a correlation-based algorithm, showing that, while still yielding sufficient results, movement reduced the accuracy of selections and negatively impacting the performance. For our study, we chose to keep all of these parameters except for distance constant and our participants stationary across all conditions to control for possible effects.

Another challenge is the Vergence-Accomodation conflict. When focusing on an object in a natural setting, the focal distance of the eye and the vergence align. While viewing a scene via a HMD however, the vergence of the users’ eyes is set to the virtual distance of the focused object behind the screen of the HMD – while the focal distance is set to the screen. This creates a mismatch which does not exist in the natural world and might lead to eye strain (7) and possibly slightly influence the individual vergence response itself (25). However, the additional gaze information along the third axis remains available over the course of the interaction in VR. Can this information be useful to improve smooth-pursuit algorithms in 3D?

While previous studies investigated the performance of smooth-pursuit algorithms in 3D VR, either correlation-based (17) or based on the Euclidian distance (27), the depth information of a third axis was not yet included in the calculations. Breitenfellner et al. (4) conclude that so far there was no extension to the existing 2D smooth pursuit algorithms for the use in VR. While Khamis et al. (17) found no significant effect of distance on the correlation-based algorithms' performance, we assume that distance will affect the performance once the third dimension is included and providing additional information to the detection algorithms.

The aim of the study was to systematically examine the potential of incorporating gaze information along the 3rd dimensional axis into the two currently most-widely used algorithms typical for 2D-smooth-pursuit interaction. One correlation-based algorithm and one distance-based algorithm were adapted to 3D. In a first experiment, the performances of both algorithms were examined by systematically varying parameters of distance and trajectory of object movement. During this experiment, only one object was visible at all times, allowing for the assessment of selection performance under ideal conditions.

The second experiment focused on the performance of the algorithms while additional objects to choose from were visible. The number of additional objects to choose from, as well as the configuration within the 3D space was varied systematically to test the algorithms under ecologically valid conditions.

The following section introduces the algorithms, followed by the methods, and a description of the virtual environment, which were used for both experiments. After that, details and outcomes of both experiments are presented individually, followed by a critical discussion and outlook.

## Algorithms and dependent variables

**Figure 1.**
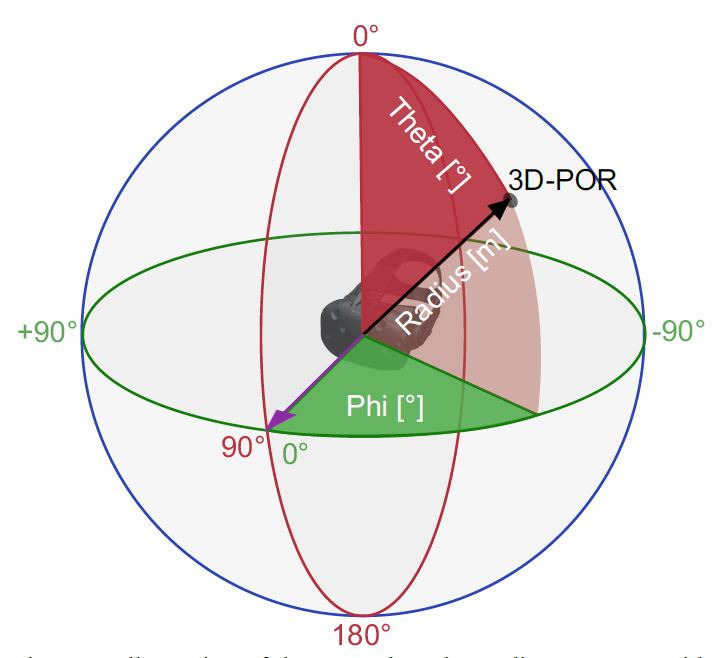
Illustration of the HMD-based coordinate system with radius r, pitch θ, and yaw φ.

While 2D smooth-pursuit algorithms often use screen coordinates to match targets and gaze, a 3D environment requires adjustments. Instead of x-, y- and z-coordinates, we defined the center of the HMD as the origin of a spherical coordinate system and matched its position to the origin of the world-space in our virtual environment. Distances were calculated as radial distance *r* with positions being defined by the radius r and the angles theta *θ* and phi *φ* for pitch and yaw respectively (Figure 1).

The 3D Point of Regard (3D-POR) was used for gaze estimation and defined as the mid-point between the respective points on the gaze vectors of each eye where the distance between both vectors reached its minimum. Both of the following algorithms were initially tested against a variable threshold. Determining the ideal threshold level for both algorithms respectively was part of experiment 1.

**A correlation-based algorithm** was adapted from the correlation-based algorithm for 2D smooth-pursuit as described by Vidal et al. (33). This algorithm calculates the product-moment correlation between gaze coordinates and the coordinates of the moving target. Instead of x and y-coordinates, the 3D-adapted algorithm uses r, *θ* and *φ* for the calculations.

**The difference-based algorithm** was based on the approach by Lutz et al. (23). The authors calculate the difference between the movement vector of targets and gaze as well as the difference in angle of the movement vectors in relation to the x-y plane. Targets are selected when both criteria fall below a selection threshold. This algorithm was adapted to 3D by using the radial distance r as well as θ and φ of the moving targets to calculate the difference to the gaze path.

### Workflow

For each new frame, first the validity of the gaze data was assessed (see Figure S1). Next, a 3D-POR was calculated and added to a Vector3-field storing the last x amount of samples, with x being defined as the size of a moving window. Upon reaching the maximum sample size, the currently oldest sample would be removed upon adding the new sample. Simultaneously, the object coordinates of each target object were stored in an identical manner in respective Vector3 fields. After updating the 3D-POR coordinates in the described manner, the respective algorithms started calculating as follows:

**The** **correlation-based algorithm** iterates over all possible interaction objects and calculates product-moment correlations between the gaze and object coordinates for each object respectively. The calculations are performed for each dimension (radius, θ, φ). In contrast to the approach in 2D, where individual correlations are compared to a threshold directly, we chose to calculate the average of all correlation coefficients for each object. While this potentially introduces an uncorrelated parameter, the effect will be the same for all respective samples which remain distinguishable via the remaining parameters. A lowering of the correlation threshold during these situations will be tested, akin to the Algorithm tested by Khamis et al. (17).

Upon calculating the correlation coefficients of all objects, the algorithm searches for the highest overall coefficient. If this correlation surpasses the particular threshold, the respective item is marked as selected by the participant.

**The** **difference-based algorithm** first splits the gaze data Vector3 field in half based on timestamps. The parameters of the halves containing the oldest and newest gaze vectors respectively are averaged. The most recent averaged gaze coordinates refer to the end point of the gaze vector, the averaged coordiantes of the other half constitute the origin of the gaze vector. By averaging the gaze data over several samples, we smooth the data and prevent obtaining false correlation values due to outliers. The endpoint of each averaged half of the Vector3 field is subtracted from the respective starting point in order to obtain a movement vector ranging from start to finish of the movement as recorded by the field interval, resulting in ${\vec{\Delta }}_{\_param}$.

These steps are performed for the gaze data as well as for the positional coordinates of each object. In order to achieve a relation between the object and gaze movement vectors, the **difference coefficients** for r, θ and φ are calculated as follows:

**(1):**
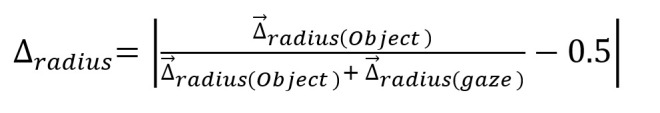


**(2):**
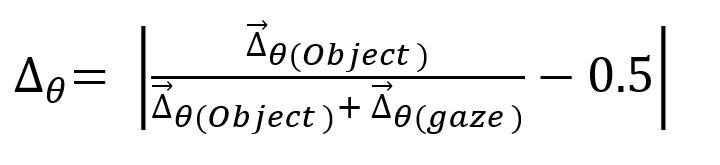


**(3):**
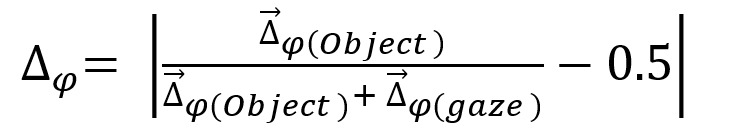


The obtained coefficients illustrate the difference between gaze radius r, gaze angle theta *θ* and gaze angle phi *φ* and the respective object parameters. The coefficients lie within the range of [0; ∞]. A coefficient of 0 indicates a perfect fit between gaze and object parameters.

**Figure 2.**
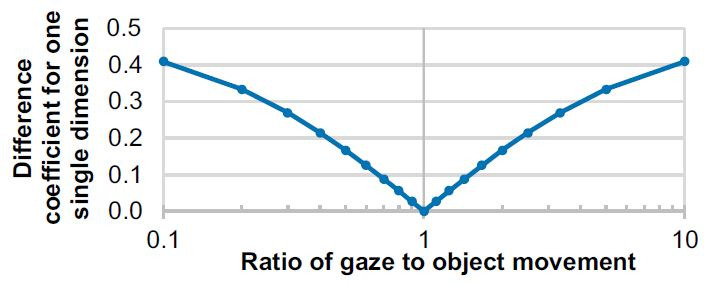
Visualization of the relation between the ratio of gaze to object movement and the resulting difference coefficient (for one dimension).

The calculated difference coefficients can be graphically expressed on a logarithmic scale based on the loga­rithm of ten. For example, if the object difference vector is kept constant at 10, a symmetrical image results for a variable gaze difference vector for positive numbers (see Figure 2). The difference coefficient would reach its minimum of 0 at a gaze vector of 10 and its maximum of 0.5 at a gaze vector of 0. Likewise, at high positive deviations, approximately 0.5 is reached. If the gaze moves in the opposite direction to the object, differential coefficients of > 0.5 are always achieved. Except in the special case that the gaze difference value should reach exactly the negative object difference value, no calculation of the difference coefficient is possible by a division by 0. This case should hardly occur practically.

In order to account for different distances and to correct the 3D POR error, the three coefficients r, θ and φ are averaged over all samples within the moving window. The algorithm then compares the sum with the threshold. The threshold level itself is adaptable and the determination of the ideal threshold level part of experiment 1.

### Dependent Variables

The following section explains the parameters that were analyzed as dependent variables in both experiments.

**Detection rate (DR)**. A true positive (TP) detection was defined by the target object surpassing the selection threshold for the respective algorithm. A false positive (FP) was defined as the algorithm detecting any other object but the currently visible one as selected. No detection (ND) took place if the threshold was not surpassed for any of the objects. The detection rate relates these parameters akin to the assessment of a binary classificator:

**(4):**
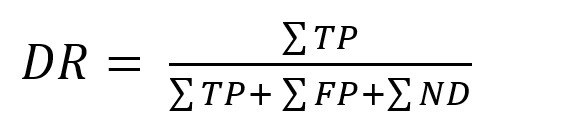


The rates of false positives (FPR) and non-detections (NDR) were calculated likewise.

**Efficiency**. The efficiency expresses the ratio of true detections to overall detections:

**(5):**
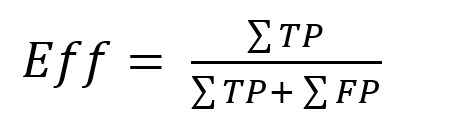


**Duration until selection**. As long durations until detections can invoke frustration in users (17), the duration until the algorithm was able to select any object was introduced as additional criterion for comparing the performance of the algorithms. The duration is expressed both in frames per second (fps) and in s.

**Further Variables**. To indicate the participants’ focus on the task, the task performance of the participants, measured as the sum of points related to the given task, and average reaction time per condition, was tracked.

## Methods

The following section describes the material used in both experiments. Differences between both settings are pointed out where applicable.

**Figure 3.**
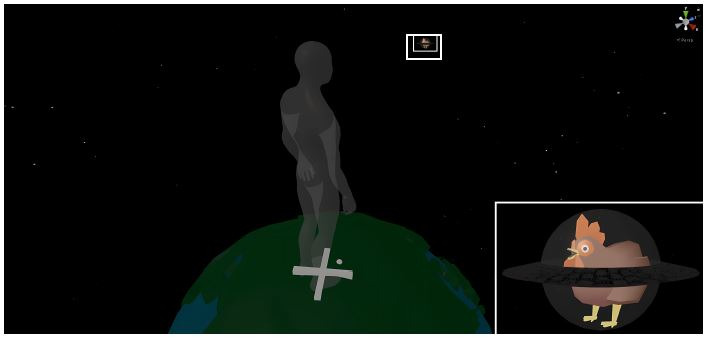
Virtual environment displaying the users' position. Lower right corner: the target object "space chicken" in a close-up.

**Virtual Environment.** A virtual environment was created with the Unity Game Engine (30). The environment is seen from the viewpoint of a person standing on a small planet of 2m diameter (38) in front of a starry sky. The environment was kept intentionally plain to reduce the influence of head movements on the task (1). An X on the planet marked the ideal position for the subjects. A light source was placed above and slightly behind the subject to prevent blinding. A chicken inside a semi-transparent spherical spaceship was introduced as a moving target (31). The target was kept visually plain to prevent sustained scanning of the details while hopefully being sufficiently entertaining to maintain subjects’ motivation. A high contrast to the backdrop was chosen to facilitate visual detection (see Figure 3). The target had a diameter of 0.07m, equaling to 10° visual angle in the close condition and 2.9° visual angle in the far condition. The size was chosen based on the results of a pre-test, constituting a compromise between identifiability over different distances and simplicity.

**Number of objects**. A maximum number of 26 individual objects being present at once was chosen in order to prevent possible ceiling effects regarding the performance of the algorithms. The high number allowed for the testing of a variety of unique movement directions within the 3D space and was therefore increased, comparing to similar studies in 2D (e.g. 37). During the first experiment, only one of the objects was visible while the others remained hidden to the user, but were taken into account during the analysis. This approach was chosen to facilitate sustained and ideal smooth-pursuit movements on one target, without other distractions. With this approach, the algorithms could be tested under an idealized, highly standardized smooth pursuit movement performed by the participants. The second experiment introduced visibility of a systematically varied number of distractors in order to retest the resulting ideal performance as it would occur “in the field” with a natural ecological validity (see experiment 2).

**Distances.** Two distances (near / far) were implemented after having been selected for optimal usability and prevention of eye strain in a pre-test. In the “near” condition the center of a spawn sphere was set to an origin at 0.4m distance (with the sphere spanning from 0.2 - 0.6m) to provide a substantial vergence of the eyes, while simultaneously being far enough to prevent eyestrain or irritation and disorientation due to too large portions of the visual field moving. The “far” condition set the center of the spawn sphere at 1.4m distance (spanning from 1.2m to 1.6m) to test the performance of the algorithm near the limit of depth detection due to parallelization of the eyes. Based on the 0.2° error as assumed for the SMI eye-tracker, this results in an error margin of 0.02m in the near condition and of 0.22m in the far condition. The distances were slightly adapted in experiment 2 (see experiment 2).

**Figure 4.**
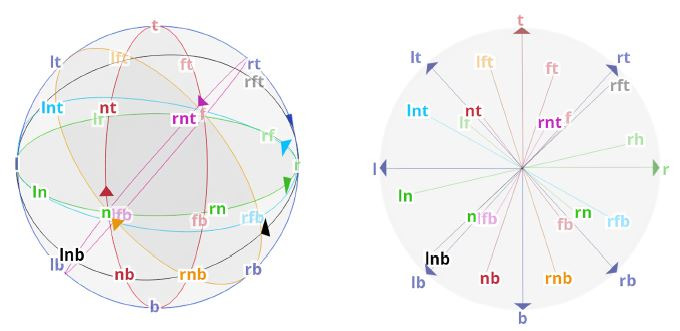
Movement patterns and arrangement of the 26 objects. The labels indicate the starting positions for circular movements (left) or the movement direction after spawning in the center for linear movements (right).

**Movement patterns.** Movement was performed in either a circular motion or in a linear motion originating from the center of the subject’s field of view. The object starting positions of the circular motions were distributed across the surface of a sphere with a radius of 0.2m, being projected from landmarks of the enclosed cube onto the sphere’s surface. Each of the eight corners, each of the mid-points between the 12 edges and the center point of each side of the cube were projected onto the sphere, resulting in 26 target spawn points overall. Seven trajectories on the surface of the sphere were determined, each containing 2-6 starting positions (see Figure 4, left). For linear motions, the object spawned in the origin of the coordinate system and moved linearly to and beyond the points described for the circular starting positions (see Figure 4, right). Velocities of each object were kept constant to minimize the occurance of potential artifacts due to anticipatory changes in pursuit movements (36).

Object velocities were set to 45°/s for the circular movement and to 0.15m/s for linear movement. The velocities in degrees visual angle were dependent on movement type and distance (see Table 1). The velocities were results of a pre-test in which we determined the usability for the subjects as well as the amount of smooth-pursuit movement as opposed to saccades (as indicators of a too fast movement) and fixations (indicating too low velocities).

**Table 1.** Overview of the spawnpoints of targets in the circular move-ment condition, as well as the directional vector for linear movement, and their velocity relative to the observer.

Movement pattern	Distance of the object group	Targets	°/s
Circular	Near	rt, rb, b, lb, l, lt	22.5
		f	15.0
		n	45.0
	Far	rt, rb, b, lb, l, lt	6.4
		f	5.6
		n	7.5
Linear	Near	rt, rb, b, lb, l, lt, t, r	21.5
		f, n	0.0
	Far	rt, rb, b, lb, l, lt, t, r	6.1
		f, n	0.0

Note: The letters indicate left (l), right (r), near (n), far (f), top (t).

**Task.** In order to provide an incentive for sustained focus on the moving target, subjects were asked to press the trigger button on the Vive Controller as soon as they detected a fogging of the space capsule surrounding the chicken to prevent it from flying blindly by clearing the fog. The reaction via the trigger button on the controller was tested in a pre-study and rated as non-distractive by users. The trigger button was specifically chosen due to being underneath the users' index finger, allowing for a quick reaction without any visual or haptic search. The fogging was timed randomly, with an average of one incident each 7.8s in the experimental blocks and of 8.4s in the practice block. A swift reaction was rewarded by an affirmative sound and the award of points (3 points for a rt ≤ 0.5s, 2 points for 0.5s < rt ≤ 1s and 1point for 1s < rt ≤1.5s). A rt > 2.5s or lack of a reaction resulted in a reduction of 3 points and a dismissive sound being played. The points were not indicated on the screen to prevent visual distraction, but participants were informed beforehand about the effects of hits and misses, and that the game would keep track of their score. After 2.5s, if no reaction occurred, the object was returned to the non-foggy state. After each block, the achieved points were displayed for the respective participant.

**Technical Setup**. We used the HTC Vive with integrated Eye Tracking by SMI (250 Hz) with a resolution of 1080 px x 1200 per eye. The refreshment rate of the screens was 90 Hz. The field of view (FOV) was 110°. The typical error of the eye tracker was 0.2° (Schiavullo, 2016). The experiments were run on an Alienware 17 R4 Laptop with an Intel Core i7 processor, 32GB RAM and a GeForce GTX 1080 with 8GB RAM. The VR-environment, run via Unity Play Mode, was displayed via SteamVR (Built May 24, 2018).

Both experiments took place in a laboratory setting. A desk was assigned at which participants filled out questionnaires testing for Simulator Sickness and assessing technical issues after the VR experience. One third of the laboratory was segmented via a cardboard divider and contained a desk with the laptop running the experiment, the VR-setup and a space of approx. 9m² for the subjects to stand freely during the interaction with the VR.

## Experiment 1

As described above, testing the reliability of the two adapted algorithms in relation to a) distance and b) movement pattern were the aim of this experiment. Furthermore, suitable thresholds for both algorithms, depending on distance and object movement patterns were to be evaluated.

### Hypotheses

**Movement Patterns**: Based on findings for 2D-experiments (33, 11) and recent findings in 3D (17) we assumed that circular movement patterns would be associated with a better performance for both algorithms compared to linear movement:

H1.1 The correlation-based algorithm performs, averaged over all distances, better on circular movement patterns compared to linear movement paths.

H1.2 The difference-based algorithm performs, averaged over all distances, better on circular movement patterns compared to linear movement paths.

**Distances**: Due to the increased estimation errors for the radius in larger distances as described in Methods, we assume a better performance at close distances for both algorithms:

H2.1: The correlation-based algorithm performs, averaged over linear and circular movement patterns, better in the near condition compared to the far condition.

H2.2: The difference-based algorithm performs, averaged over linear and circular movement patterns, better in the near condition compared to the far condition.

**Interaction**: We assume that the impact of increased eye tracking errors in larger distances and its’ effect on the calculation of the radius is inequal for linear and circular movement patterns due to the different proportion the radius calculation has for the overall algorithm:

H3.1: The change in detection rate (DR) between the near and the far condition differs between circular and linear movement patterns for the correlation-based algorithm.

H3.2: The change in DR between the near and the far condition differs between circular and linear movement patterns for the difference-based algorithm.

No previous assumptions were made for the optimal detection threshold level to be used for the algorithms. Instead, the threshold levels (TL) were analyzed iteratively to find the optimal threshold for each algorithm. The comparison of the performance levels of both algorithms to each other were of interest as well. Due to the multitude of possible factors influencing the performance, no general hypothesis about the superiority of any of both algorithms was stated beforehand.

The task performance, as indicated by the amount of points received in the detection task, was used as indication of the attention users directed to the interaction, and with that, served as an indicator of the quality in which the smooth-pursuit task was performed.

### Experiment plan

The experiment encompassed one practice block, four experimental blocks and one additional block. The timespan of initiating and completing one singular object movement was defined as one trial. One object movement translates to the spawning of the target object, the space-chicken remaining at rest for 1s and then moving along one of the pre-defined paths for 4s.

The practice block contained four trials with linear movement patterns in a pre-defined order and prolonged movement durations (20s), taking approx. 1.5 minutes in total. The subsequent four experimental blocks were presented latin square randomized and contained 78 trials each. Each block represented one combination of the independent variables (near/circular, near/linear, far/circular, far/linear). The 78 trials were presented in three rounds, with each round presenting all 26 possible object variations of the respective condition in a randomized manner. Each block had a duration of 6.5 minutes.

The additional block displayed a variation of the movement patterns and collected data for another research question and will be discussed in a different work. It took 7.5 minutes to complete.

### Experimental procedure

Subjects: N = 12 participants (6 ♀, 6 ♂) aged between 23 to 30 years (M = 27.4, SD = 1.8) took part in the first experiment. Five persons had corrected to normal vision, with three using contact lenses and two taking part without corrective measures (-1 and -2 dpt). Half of all participants were novice to interacting with a VR environment.

Participants were greeted, prompted to read the participant information informing about the procedure and voluntary nature of the studies. Upon agreement, they filled out a demographic questionnaire and were then handed the instructions for the trial. If no questions remained, they put on the HMD and were assisted if necessary. They were then handed a Vive Controller to be used with their dominant hand. The controller was not depicted within the VR. Participants were standing during the whole experiment.

Participants were then asked to physically walk onto the marking of a cross on the planet and turn until they were facing an orientation dot visible in front of them. A 5-point calibration was then performed. Afterwards, they could start the practice block via a press on the side button of the controller. Then, they were asked if they had any questions, and if none remained, the five blocks were started and run in the afore described randomized manner.

Afterwards, they filled out surveys assessing Simulator Sickness and technical issues. Upon completion, the participants were thanked, compensated with student credit hours if applicable, and snacks if not, and were then given the option to ask questions about the experiment and to receive a detailed description of the experiments’ purpose.

### Analysis

The gaze data was obtained using the SMI Unity plug-in. The 3D POR was calculated using an adaptation of the Math3d class (19). The product-moment correlation was calculated using the Math.NET Numeric library (28). The algorithms were implemented within the Unity development environment, using C#. All further analyses for experiment 1 were done offline.

The time window and starting point for the algorithms were specified based on previous literature. For the correlation-based algorithm recommendations ranged from 0.5s with ~20 data points (33) to 1s with ~30 data points (11). Our goal was to achieve an optimal spot between a high number of data points, increasing the detection rate, and a short time frame, lowering reaction times for later online use (11, 17). We chose to include 40 samples into the testing window. With an average fps of 60Hz as measured in a pre-test, this resulted in a duration of 0.67s. The same time window was implemented for the difference-based algorithm.

Thus, analyses started 40 frames after the onset of movement.

**Calculation of the optimal threshold**. The optimal threshold (OT) for both algorithms was tested iteratively. For the correlation-based algorithm, we iterated over thresholds of correlation values between 0 and .95 in steps of .05. For the difference-based algorithms, we iterated over thresholds between .20 and .01 in increments of .01, resulting in 20 data sets with linearly increasing thresholds for each algorithm.

Efficiency, TP, FP and ND were each averaged over all trials for the respective algorithms. The residues for the data sets with the threshold resulting in the best performance were tested for normal distribution via QQ-Plots and a subsequent Shapiro-Wilk Test and then further analyzed via an Analysis of Variance.

To gain further insights into the non-significant results we performed a sensitivity analysis with G*Power.

For directional analysis, the DRs of each of the 26 linearly moving objects were calculated for the near and far condition. Afterwards the DRs of the objects moving in one of the six base directions (left, right, up, down, nearing, distancing (far)) were averaged. Thus, per base direction the DRs of nine objects were averaged.

**Comparison of the algorithms.** We employed a sign test to compare the performance of the two algorithms (3). This non-parametric test allows for a comparison on trial-level. Therefore, both data sets were combined, depicting the results of both algorithms (TP, FP, ND). All entries with equal decisions were omitted, leaving only the rows with diverging entries. For the remaining trials, a “+” or “-“ was assigned, depending on a correct or incorrect decision made by the respective algorithm. The sum of the resulting “+”s was calculated for each algorithm and a Binomial test was performed, testing if the likelihood of a “+” appearing was distinct from the likelihood or random appearance (p = .5).

### Results

**Hypotheses**. The ANOVA yielded a confirmation of the main effect for movement type for the correlation-based algorithm (F(1,11) = 24.27, p < .001, η² = .29), but not for the difference-based one (F(1,11) = 0.98, p = .344, η² = .01), confirming H1.1 and refuting H1.2. A main effect for object distance was discovered for both the correlation-based algorithm (F(1,11) = 190.77, p < .001, η² = .75) and the difference-based one (F(1,11) = 148.20, p < .001, η² = .42), confirming hypotheses 2.1 and 2.2.

**Figure 5.**
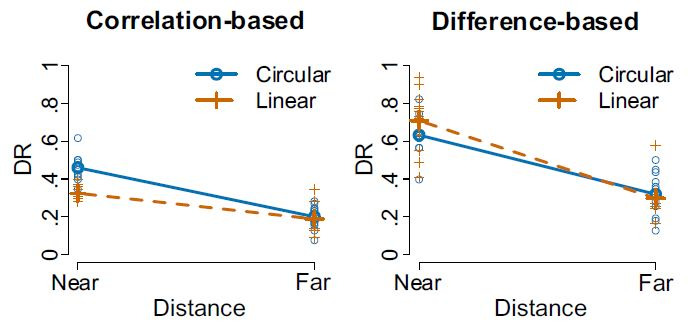
Interaction Plots for the correlation-based (left) and difference-based (right) algorithms for close and far distances, in interaction with the movement type (circular or linear).

Interaction effects for distances x movement types were found for both the correlation-based algorithm (F(1,11) = 9.00, p = .012, η² = .22) and the difference-based one (F(1,11) = 5.94, p < .033, η² = .01), see Figure 5, confirming H3.1 and H3.2.

As the main effect concerning movement type for the difference-based algorithm was not significant we performed a post-hoc sensitivity analysis. With the sample size of N = 12, the test would have revealed effects of at least η² = 0.09 with a probability of .90. Thus, for hypothesis H1.2, we assumed that there was either a very small effect or no effect.

**Figure 6.**
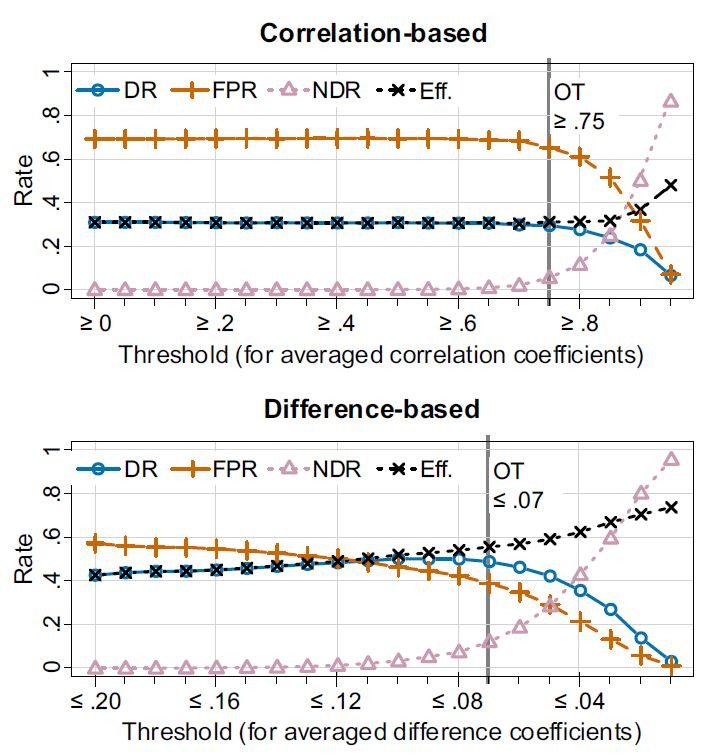
Performance graphs for the correlation-based (top) and difference-based algorithm (bottom), based on detection rates, false-positives, non-detections and efficiency averaged over all trials. The chosen threshold level (OT) is indicated by the respective vertical line.

**Optimal threshold**. The correlation-based algorithm presented its’ best detection rate (*M* = .31, SD = .46) at a threshold interval of between .65 to .80 (*M* = .28, SD = .45) with a rapid decrease of detections for higher thresholds. The rate of false detections has a maximum at a threshold of .69 (SD = .46) and decreases for thresholds ≥ .75 (*M*= .65, SD = .48). The ND rate remains *M* = 0 (SD = 0) for lowest thresholds and remains low for thresholds between .45 to .65 (*M* < .01, SD <.10). At a threshold of .70, the ND rate begins to increase (*M* = .02, SD = .14). The Efficiency follows the curve for the detection rate up to the threshold of .70 where first NDs take place. We selected an optimal threshold of .75 for the correlation-based algorithm which equals an Efficiency value of .31. After that point, the FP rate begins to decrease while the detection rate remains close to its’ maximum. The average detection time from the beginning of the movement is 1.18s (SD = .69) or *M* = 70 frames (SD = .41), see Figure 6 (top).

The difference-based algorithm shows a pattern similar to the correlation-based algorithm. The detection rate increases from the initial .20 up to a threshold of .10 (*M* = .50, SD = .50), followed by a decrease. The rate of false detections sinks continuously while the ND rate remains less than or equal .05 until it increases rapidly for thresholds smaller than .08 (*M* = .08, SD = .03). The Efficiency curve is similar to the detection rate for thresholds larger than .10, but further increases while reaching a maximum at the most restrictive threshold of .02 (*M* = .74). A threshold of .07 was selected as optimal, as it resulted in, all parameters combined, the best overall performance (see Figure 6, bottom).

Therefore, the thresholds of .75 for the correlation-based algorithm and of .07 for the difference-based algorithm were used for all following comparisons.

**Comparison of the Algorithms**. Averaged over all trials, the difference-based algorithm achieved a higher DR compared to the correlation-based algorithm (*M* = .49, SD = .49 vs. *M* = .29, SD = .49). The Binomial test revealed a significantly higher DR of the difference-based algorithm compared to the correlation based one (p <0.001, with 1124 of 1516 trials showing a higher DR for the difference-based algorithm).

**Figure 7.**
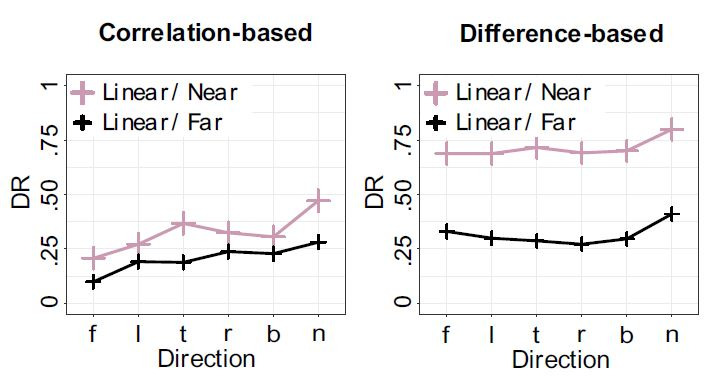
Detection performance of the two algorithms for linear movements averaged over the six basic directions towards the end point of the movement vector (left (l), right (r), top (t), bottom (b), near (n), far (f)).

**Directional movements of the objects**. The performance of the correlation-based algorithm showed the lowest averaged DR for objects that performed a linear movement along the vector f (“far”) away from the observer in both the near (*M* = .21, SD = .08) and the far condition (*M* = .10, SD = .08). Objects with a linear movement along the vector n (“near”), approaching the observer, yielded the highest averaged DR (near *M* = .47, SD = .10, far *M* = .28, SD = .09). The difference-based algorithm showed the highest averaged DR for objects with a linear movement towards the observer (vector n) as well, with considerably higher averaged DRs (near *M* = .80, SD = .07, far *M* = .74, SD = .20, see Figure 7).

**RT-Task.** On average, 138.46 (SD = 8.24) of 150 achievable points per condition were scored by the participants. No subject achieved less than 116 points (77%) in any condition. With 50 reaction stimuli per condition, 2400 reaction stimuli occurred across all trials, with 2385 of them (99%) being responded to by the participant within 2.5 s. The average reaction time was 0.44 s (SD = 0.16).

### Discussion of experiment 1

As predicted, the correlation-based algorithm showed a better performance for circular movements compared to linear movement patterns (H 1.1). This difference was not established for the difference-based algorithm, which showed no significant difference between both movement types, with a sensitivity test suggesting either no or a very small effect (H 1.2). This ties into the overall higher DRs of the difference-based algorithm that were obtained across all trials. The exploratory analysis of the influence of directional movements suggests a higher robustness towards linear types of movement for the difference-based algorithm. Interestingly, the difference-based algorithm performs highest for movements along the z-axis, i.e., towards or away from the observer, in which the rate of change would be lowest.

Overall, both algorithms performed better if objects were shown within a close range compared to displaying the objects in larger distances, independent of movement patterns (H2.1 and H2.2). Movements virtually closer to the eyes of the observer benefit from a lower estimation error, which accumulates along the third axis. Furthermore, the further away the movement, the smaller the visual distance covered. Combined with additions of estimation errors, inaccuracies increase. The confirmation of the interaction effect between distance x movement type (H3.1, H3.2) supports this assumption. The higher DRs in closer distances would suggest adopting a design principle in which it was recommended to set stimuli to be selected via smooth pursuit within the near plane of the virtual environment. However, only one object was shown at all times. While this was done to create ideal conditions for sustained smooth pursuit movements, it also created an artificial setup which kept eye strain due to different visible stimuli at minimum. One of the goals of the second experiment therefore was to evaluate in a pre-study if the addition of further visible objects would have any adverse effects on the observer.

The high rates for successful reactions of participants during the reaction task indicate sustained attention towards the object. Together with the aforementioned display of one singular visible stimulus (with the other 25 stimuli being represented mathematically), ideal conditions for testing the algorithms were created. The optimal threshold level for both algorithms were therefore selected within a setup that allowed to test the optimum performance. How would the achieved results hold up under ecologically more valid conditions? To answer this was the scope of experiment 2.

## Experiment 2

Having identified an optimal threshold level for both algorithms under artificially optimal selection conditions, we could now test the performance under a systematically varying number of visible objects for the participant to choose from. Due to the interaction effects of distance and movement types found in experiment 1, experiment 2 included these parameters, too with the aim to a) test performance levels of both algorithms under a selection of typical setups that might be present in VR applications using smooth-pursuit object selection and b) ideally allow for recommendations of the maximum numbers of objects, movement types and distances, including performance data as well as user feedback.

Additionally, based on the findings of experiment 1, we assume that the optimal threshold level for detections by each algorithm varies, depending on the interaction of distance, movement pattern and, as introduced in this section, the number of objects present. We aimed to derive a formula that could indicate the optimal threshold for each algorithm under these varying conditions, taking into account the rate of non-detections, detections, and false positives.

### Adjustments to the experimental setting

**Figure 8.**
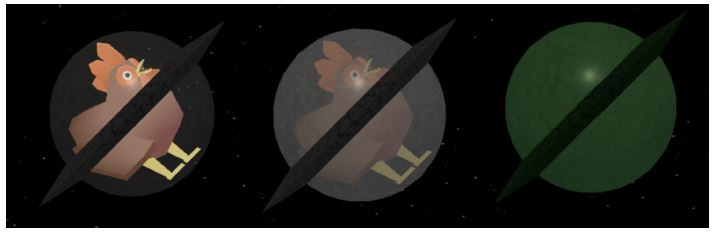
Left: target object; Middle: clouded target object while reaction time task; Right: non-target.

The main difference to experiment 1 is the presence of non-target objects: objects in varying quantity that moved within the same plane of distance and movement pattern as the target object. These non-targets kept the shape of the spherical shape ships but were colored in green and lacked the target chicken (see Figure 8).

**Number of visible objects**. In order to determine the number of non-targets to be tested, the logfiles of experiment I were re-evaluated. Subsets of the original 26 objects were created, iteratively reducing the number of objects taken into account for the gaze-object-comparison by the algorithms. DRs were calculated for these subsets to determine the performance of the two algorithms for different amounts of objects. Based on these re-evaluations, the number of distractors for experiment II was set to an interval from two to eleven, resulting in a maximum object count of twelve, including the target object. An object count of less than three was likely to generate ceiling effects, reducing the informative value of these conditions. More than twelve objects displayed at the same time would create a substantial amount of overlap between objects in the start position or during the object movement within this setup and were therefore excluded.

**Table 2.** Overview of object configurations and their respective spawn points (circular) or target point of movement (linear).

Object config.	spawn point of objects (circular) / endpoint of movement vector (linear)
3 A	lnt, rnt, f
3 B	lnb, t, rfb
4 A	lt, lb, rb, rt
4 B	lnb, rnt, lft, rfb
5 A	l, b, r, t, f
5 B	lnb, rnt, lft, rfb, f
6	lnb, l, b, r, t, rft
7	lnb, rnt, l, b, r, lft, rfb
8 A	l, b, r, t, lft, lfb, rfb, rft
8 B	lnt, lnb, rnb, rnt, lf, fb, rf, ft
9 A	lt, l, lb, b, rb, r, rt, t, f
9 B	rnb, rn, rnt, lt, l, lb, ft, f, rb
10 A	lnt, lnb, rnb, rnt, t, b, lf, fb, rf, ft
10 B	nb, nt, lt, l, lb, rb, r, rt, ft, fb
11	rnt, rn, rnb, lt, l, lb, b, t, rfb, rft, f
12 A	lnt, lnb, rnb, rnt, l, b, r, t, lft, lfb, rfb, rft
12 B	ln, nb, rn, nt, lt, lb, rb, rt, lf, fb, rf, ft

Note: abbreviations: left (l), right (r), near (n), far (f), top (t), bottom (b). See Figure 4 for the spatial distributions of points.

The spatial distribution of the non-targets was created by omitting objects from the original invisible, but simulated 26 objects. The remaining configurations were distributed evenly across the volume extending in front of the participant (see Figure 4). In total, 17 different object configurations were tested, including ten different object counts (3-12), of which seven were tested in different arrangements (A/B, see Table 2).

**Distances**. The distance of the “near” condition was adjusted to 0.8m (before: 0.4m) because pre-tests with the adapted number of visible objects revealed a high eye-strain for the participants, due to having various objects in their immediate field of view in such a close proximity. This adjustment limits the maximum vergence of the eyes and with that reduces the range of additional information added to the algorithm on the third dimension, but was decided to be necessary to ensure optimal and strain-free conditions for the participants. Due to the distance of the “far” condition having been set to 1.4m in experiment 1 in order to test the maximum viable distance, this distance was not increased further. With the radius of the sphere on which the spawn points were distributed remaining at 0.2m, this results in effective spawn distances of 0.6-1.0m (centered at 0.8m) in the near condition and 1.2m to 1.6m (centered at 1.4m) in the far condition. Further implications are discussed in the overall discussion.

**Task**. The feedback from pre-test participants led to the adjustment of the point-system in the reaction-time task. To prevent demotivating the participants by a low score at the end of each experimental block, wrong reactions only led to a point reduction of -1 in experiment 2.

**Object visibility duration**. The objects were shown immobile for 2 seconds after spawning before starting their movement, which continued for 4 seconds. The time of visibility in the initial resting phase was prolonged to 2 seconds (before: 1 second) to allow enough time for identification of the target. Hence, the overall duration of the experiment slightly increased.

**Starting position of objects in linear conditions**. While the target object was rendered directly in the center of the visual field in experiment 1, the presence of various objects in experiment 2 required a slight adjustment of the start position to avoid overlap. The objects started slightly set off from one another, each slightly moved in the respective direction of the following movement.

### Hypotheses

Based on the previous results, we derived the hypotheses for experiment 2 as follows:

Movement pattern

H1.1 The correlation-based algorithm performs, averaged over all distances, better on circular movement patterns compared to linear movement paths.

H1.2 The difference-based algorithm performs, averaged over all distances, equally for circular movement patterns and linear movement paths.

Distance

H2.1: The correlation-based algorithm performs, averaged over linear and circular movement patterns, better in the near condition compared to the far condition.

H2.2: The difference-based algorithm performs, averaged over linear and circular movement patterns, better in the near condition compared to the far condition.

Number of non-targets

H3.1: The correlation-based algorithm performs better the fewer objects are present.

H3.2: The difference-based algorithm performs better the fewer objects are present.

Comparison of algorithms

H4: The difference-based algorithm performs, on average, better than the correlation-based algorithm.

Further research questions:

Optimal thresholds. We aimed to derive a formula for the OT, integrating the different performance parameters (DR, FP, ND). Selection time: As the aim of this study is to facilitate the application of online smooth-pursuit-based selection in 3D VR, the reaction times of both algorithms were recorded. No hypotheses were stated regarding a possible impact of movement pattern, distance and object count on the selection time of both algorithms. Instead, the reaction times across all conditions were tested exploratively. To allow for design recommendations from the users’ point of view as well, the preference of participants for movement types as well as object distance and numbers were assessed.

### Experiment plan 

The experiment consisted of one practice block and four randomized experimental blocks. As in experiment I,, one experimental block represented one unique combination of object distance (near/far) and movement pattern (linear/circular). In each of the experimental blocks, the number of displayed objects progressively increased from three to twelve. Since some of the object numbers were tested in two different arrangements, the resulting 17 objects variants (see Table 2) were completed one after the other. Each of these object variants were repeated three times in a row, with an object selected by random as the target object. One trial consisted of a 2 second phase, where the objects were presented in the center of the visual field, followed by a 4 second phase of object movement.

In total, 51 trials constituted one experimental block, resulting in 204 trials in total. Each block took 5.1 minutes to complete.

**Participants**. N = 30 participants (15 ♀, 14 ♂, 1 diverse) aged between 20 to 35 years (*M* = 26.6, SD = 3.7) took part in the second experiment. 13 reported corrected to normal-vision, with six individuals using contact lenses, five glasses and two without corrective measures. The test procedure was identical to experiment I.

### Analysis

The dependent variable mainly used for the analysis was the detection rate DR, which is defined as the proportion of true positive detections from all trials (see Formula 4). The analysis process was equivalent to experiment 1 regarding 3D POR calculation and the moving window of 40 frames for calculation.

An ANOVA was performed to test the impact of varying object numbers on the DR under the four test conditions (near/linear, near/circular, far/linear and far/circular) for both algorithms.

For each object configuration, an optimal threshold was determined by using the same method as described in the previous experiment. For the correlation-based algorithm, we iterated over thresholds between 0 and .95 in increments of .05. For the difference-based algorithm, values between .20 and .01 were tested in steps of .01.

The configuration yielding the optimal threshold for the respective object variant was further analyzed. In order to perform an Analysis of Variance to test H3.1, the data was tested for normal distribution of the residuals with a Shapiro-Wilk-Test and a Mauchly-Test for sphericity. Due to the amount of data, exemplary object counts (with 4, 6, 8, 10 and 12 objects) were tested for their specific main effects.

Since the data of some of the tested object configurations violated the normal-distribution of residuals, additional QQ-Plots were used. Based on Lix, Keselman & Keselman (22), who refer to the ANOVA as robust regarding violations of the normal distribution of residuals and on Villasenor et al. (34, p. 1874) who say, that the Shapiro-Wilk-Test is “too strict”, a two factor ANOVA was chosen as test measure, although not all requirements were met.

To test H4, the selected object counts of 4, 6, 8, 10 and 12 objects were each individually tested for the differences in detection rates between the correlation-based and difference-based algorithms. The requirements to perform a *t*-test include normal distribution of the difference variable. Since this was not given and the *t*-test is more vulnerable to undesired impacts, the non-parametric test alternative Wilcoxon-sign rank test was performed.

We investigated if an ideal threshold could be determined for each object configuration. We therefore defined a formula for Efficiency_1 based on their DR, FP and ND. In terms of application, ND are preferred over FP because the trial can be repeated while corrective measures needed to be taken for a false selection. More FP are likely to have a worsening impact on user satisfaction. The formula used is:

**(6):**
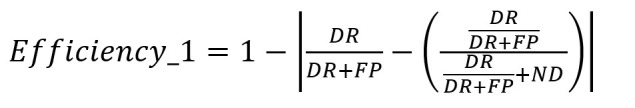


$\frac{DR}{DR+FP}$ represents the proportion of correctly detected objects (DR) out of all the trials where an object exceeds the pre-defined threshold – in the following, this term will be referred to as “effectiveness”. In the second part of the term, the ND rate is additionally taken into account, calculating the proportion of the “effectiveness” out of all trials. The absolute value of the “effectiveness” was used and subtracted from 1.

This formula reaches its’ maximum value when the ND exceeds the FP, favoring repeats over false selections. The formula therefore is able to determine for which range of threshold values the FP are decreasing, which is desirable.

Efficiency_2 is also based on what we previously defined as “effectiveness” ( $\frac{DR}{DR+FP}$) and the ND, but focuses on the rate of change from one threshold value to the next.

**(7):**
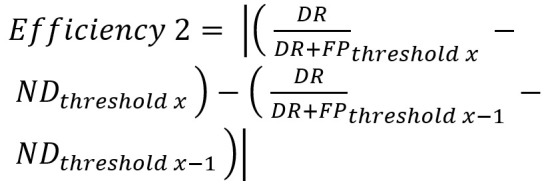


With this approach we investigated if a rapid increase in ND and decrease in DR emerged, indicating a specific threshold as ideal, as represented by a high slope of the graph for Efficiency_2.

### Results

**Movement pattern and distance.** On average, circular object movements resulted in a trend towards a higher DR for both algorithms (H1.1/1.2), but the main effect only surpassed the significance threshold for 4 and 6 objects for the correlation-based algorithm (p = .003, $\eta_{g}^{2}=.06$ and p = .001, $\eta_{g}^{2}=\;.13$) and for 4 objects for the difference-based algorithm (p = .043, $\eta_{g}^{2}$=.03). We hypothesized an effect for all object counts for the correlation-based algorithm but none for the difference-based algorithm. The general tendency suggests that the difference-based algorithms had higher DRs for circular movements, but for both algorithms the differences in DRs were lower than in experiment 1.

The impact of distance was found to be much smaller than in experiment 1. Only for the trials with all 12 objects visible, both algorithms performed better in the “near”-condition (corr.-b. A. p = .004, $\eta_{g}^{2}=.08$, diff.-b. A. p = .021, $\eta_{g}^{2}=\;.04$), which is compliant with H2.1 and H2.2. Additionally, the correlation-based algorithm detected more objects in the near-conditions in the variants with 8 objects (p = .027, $\eta_{g}^{2}$=.03).

**Object count.** The ANOVA yielded a confirmation of H3.1 and H3.2, regarding the main effect of object count for both the correlation-based (p < .001, $\eta_{g}^{2}$ = .61) and the difference-based algorithm (p < .001, $\eta_{g}^{2}=\;.27$). Additionally, an interaction effect between the object counts and the test condition was found to be significant for the correlation-based algorithm (p = .007), but only with a comparably low effect of $\eta_{g}^{2}$ = .05.

**Figure 9.**
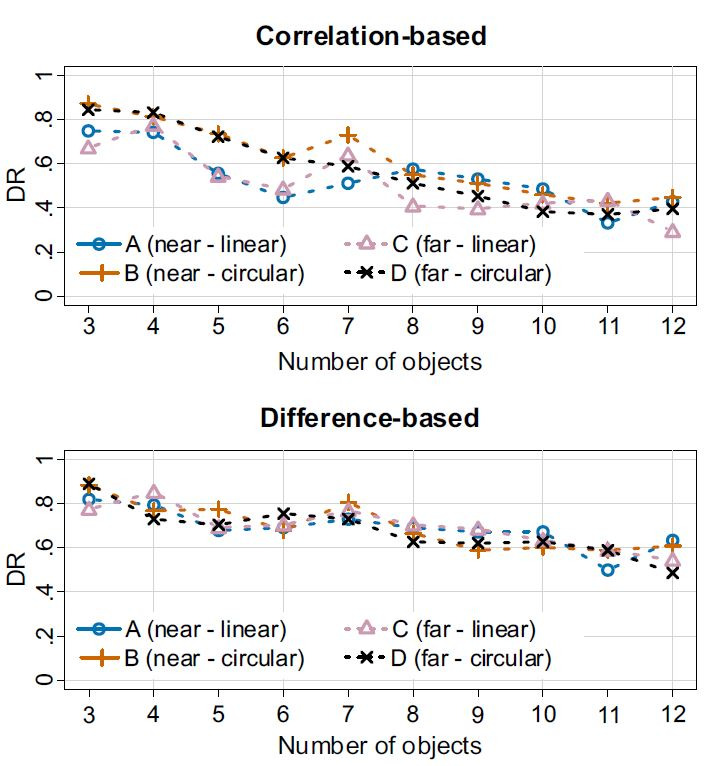
Detection rates of both algorithms for movement patterns (linear, circular) distance (near, far) and number of visible objects.

**Figure 10.**
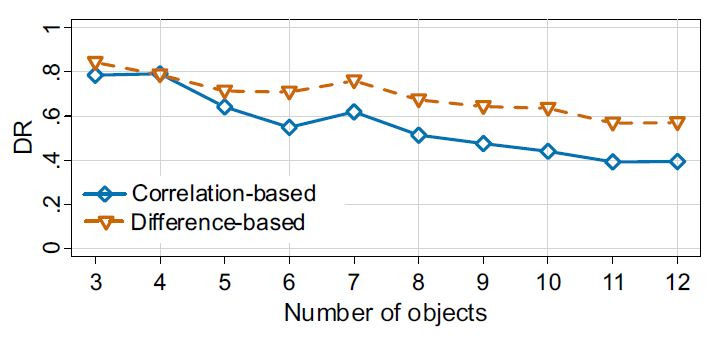
Detection rates of both algorithms for all numbers of simultaneously visible objects, averaged over all experimental conditions.

**Comparison of algorithms.** The difference-based algorithm outperformed the correlation-based algorithm in nearly all of the tested trials, in alignment with H4 (see Figure 10). Four out of the five selected object counts for further investigation (4, 6, 8, 10 and 12 objects) resulted in higher detection rates for the difference-based algorithm (4 objects: p= .418, r= .02; 6 objects: p= <.001, r= .48; 8 objects: p= <.001, r =.41; 10 objects: p= <.001, r= .58; 12 objects: p= <.001, r = .35).

**Optimal threshold.** The difference-based algorithm produced a lower FP rate than the correlation-based one. Therefore, the maximum value of Efficiency_1 is achieved at a comparatively low threshold value (see Table S1; e. g. .13 for object variants with 3 objects; .1 for object variants with 6 or 9 objects; .06 for object variants with 12 objects). For the correlation-based algorithm, which has on average a higher FP rate, the maximum of Efficiency_1 is achieved for higher thresholds, therefore leading to more conservative selections. Using the Efficiency_2 formula, optimal thresholds for most numbers of visible objects for the correlation-based algorithm were identifiable due to the impact of increasing DR and decreasing ND. For the difference-based algorithm, Efficiency_2 painted a less clear picture, as NDs were already low.

**Table 3.** Overview of durations for and detections for both algorithms, averaged, and for exemplary numbers of objects.

objects	correlation-based		difference-based	
	select.	detection	select.	detection
*M* (all)	1.23	1.18	1.53	1.53
3	1.03	0.99	1.12	1.10
6	1.05	0.98	1.26	1.33
9	1.34	1.30	1.62	1.62
12	1.13	1.05	1.54	1.61

Note: Duration is provided in seconds. Selections are defined as true positives and false positives. Detections are defined as true positives only.

**Detection time**. Overall, the correlation-based algorithm performed selections (including true and false positives) after an average of 1.23s, averaged over all conditions (see Table 3). If only detections (true positives) were considered, the duration shortened to 1.18s. In contrast, the difference-based algorithm needed 1.53s for selections as well as true positive detections and showed an overall higher time for both selections and detections.

**Subjective results**. 17 out of 26 participants indicated, across all conditions for movement type and distance, that on average, seven objects were the most comfortable to interact with (*M* = 7.18, SD= 1.67, “What number of objects was the most comfortable for you?”). The remaining nine participants, were comfortable to interact with any number of objects between three and 12. Asked which number of objects were too many to complete the primary task unhindered, the majority (18) of participants indicated that there were no hindrance for the maximum number of objects shown simultaneously (12). Participants voiced that the clear visual distinction between target and non-targets helped in completing the task. Eight participants felt disrupted by the increasing number of objects at an average threshold of *M* = 9.86 (SD = 1.55) objects.

No clear preference for either linear (favored by 11 participants) or circular movement types (favored by 10) emerged. Five participants had no preference at all. Asked why they preferred their chosen type of movement, the reasons for linear movement were stated, with number of participants in braces, as “high predictability of continuation of the movement” (4), “less coverage between objects” (4), and “less straining for the eyes” (1). For circular movement, the reasons were “less eye movement needed/less visual angle covered” (5), “aesthetics of the movement pattern” (3), “better resolution of the objects on the HMD” (2) and “ease of interaction” (1).

A preference for the far display condition (15 participants) over the near display of objects (6 participants) emerged. Five participants indicated no preference. Reasons for favoring the far distance (1.4m) were “less head movement needed” (6), “better overview” (5) and “less eye movement needed/smaller visual angle covered by the objects” (4), but also “a better resolution” (1). The near condition was favored due to “a better resolution” (3), for “no particular reason” (2) or because of the “bigger object size” (1).

## Discussion

**Performance.** Across both experiments, the difference-based algorithm provided a better performance compared to the correlation-based one, if performance is operationalized by high detection rates. However, as experiment 2 has shown, the higher reliability comes at a cost, as both overall selection times and detection times were slower, compared to the correlation-based algorithm. The latter, on the other hand, provides faster interaction for the user. The impact of different movement patterns was higher for the correlation-based algorithm as well, with circular types of movement increasing DRs for this algorithm, but not for the difference-based algorithm which performed more homogenously across both conditions.

**Distances.** While experiment 1 showed a clear advantage in detection for close distances (0.4m) for both algorithms, experiment 2 could not establish a significant difference in performance between both conditions. This might have been due to the adaptation of distance in the near condition, setting the spawn point to 0.8m. This was required by the results of pre-tests after introducing additional visible objects. It is very likely that the decrease in distance between both near and far condition reduced differences in detection rates, as the original distance between both conditions was nearly halved. Furthermore, both distances were indicated by the center of a sphere on the surface of which objects could spawn, adding a radius of 0.2m., resulting in a distance of only 0.4m between spawn points on the back side of the sphere in the near condition (centered at 0.8m) and spawn points on the front side of the sphere (1.2m, centered at 1.4m) in the far condition. However, the rationale behind the exact location of the spawn center in the far condition was to test the limits of the algorithm and the usefulness of including the third axis into calculations close to a point where parallelization of the eyes would hinder depth detection. While trends towards better detection in the near condition were still detectable, experiment 2 showed that both algorithms work reasonably well within both distances.

**User Experience.** The adaptation of spawn distances took place because the introduction of additional objects in close proximity caused eye-strain that did not occur while only one object was present. We assume that the increased amount of movement in a comparatively large portion of the visual field had contributed to the discomfort. After experiment 2, individuals who preferred the far condition indicated that they liked not having to perform large eye movements, further supporting that assumption. This ties into a limitation of the usefulness of the third spatial axis for any kind of gaze analysis compared to the other axes: not all distances are equally feasible, depending not only on physical and technical limitations but also on the number of stimuli present in the visual field. Drewes et al. (10) have demonstrated that user preferences corresponded to optimal detection rates in 2D smooth pursuit tasks with constant target velocities. We therefore assume that, by having adapted the speed to the users' preferences in our prestudy, the chosen speed parameters would approach optimal settings to investigate the algorithms performances. In 3D, the perceived speed of the targets might vary due to targets moving away or towards the user. We encourage further researching the relationship between user preference and detection rates specifically when including the third dimension.

**Number of visible objects.** While experiment 1 investigated the optimum performance of both algorithms under ideal conditions, the introduction of visible objects allowed to test both algorithms in an ecologically more valid setup. Hypotheses H3.1 and H3.2 of experiment 2 were confirmed, showing that smaller numbers of objects increase the performance of both algorithms. The addition of feedback from participants allow to balance affordances of the algorithm with user preferences. While not all participants showed a preference for specific object numbers, the majority did and indicated that seven objects were preferable. Additionally, we found that for object counts ≤ 6 the FP rates of the difference-based algorithm were lower than the ND rates. This is desirable, as trials without a detection can be repeated, while trials with a false detection can lead to a worse user experience and increase the overall interaction time. We therefore recommend limiting the number of simultaneously shown objects to choose from via smooth-pursuit to 6, and suggest to not surpass a number of 9 objects at a time, as this was indicated as being perceived as too much by almost a third of participants.

**Number of participants**. For this study, two experiments were conducted. The first experiment was to test both algorithms under ideal conditions with only one target being shown to a comparatively low number of twelve participants, but with a high number of trials. To account for this number, a sensitivity analysis was performed for the non-significant results, revealing that effects of at least η² = 0.09 would have been revealed with a probability of .90. The second experiment introduced an ecologically valid variation in the number of visible objects as described above and was therefore considered as much closer to real-world applications, which is why we allocated a comparably higher number of 30 participants to this experiment.

**Calibration.** One of the great advantages of smooth-pursuit based interaction is the option to be used for spontaneous interaction without (33) or only minimal (23) calibration. While we aimed for ecological validity in the second study, we still included a calibration for this experiment to control for potential sources of error. The additional calibration time was feasible due to the experimental conditions, but might pose a hindrance for applications. We suggest to compare using different calibration procedures, e.g., smooth-pursuit based (e.g. 26, 2) or regression-based (9) and calibration-free performances to provide further references for application.

**Optimal threshold.** The idea to introduce a formula to indicate the optimal threshold provided helpful support in threshold selection, but ultimately needs more fine tuning. For both algorithms, the Efficiency_1 tends to favor more conservative thresholds when a higher number of objects is shown (> 6 objects). This inherently results from trying to prevent false positives, of which the likelihood to occur increases with each additional distractor, as the distance to other selectable objects is reduced. In most cases, this conservative threshold results in DRs close to the maximum of the algorithm. However, we suggest an adjustment for object counts larger than six, as detection loss might otherwise occur.

The approach of calculating the optimal threshold seems promising, but not perfect. For some object variants, Efficiency_2 failed to suggest a clear threshold as a steady slope emerged, with no significant maximum. This occurred more often for the difference-based algorithm than for the correlation-based algorithm. As the difference-based algorithm was generally more reliable than the correlation-based algorithm, the lower variance of DRs and NDs led in turn to a lower change in Efficiency_2. For our analyses, we used a hybrid approach, taking into consideration the thresholds suggested by Efficiency_1 (for the difference-based algorithm) and Efficiency_2 (for the correlation-based algorithm) and a visual inspection of the development of DR to determine individual thresholds for each of the tested object configurations. The aim was to choose a threshold which would produce a high DR and prefers ND over FP. However, this threshold was selected manually. A further development would be the further refinement of the developed formulae, and in a second step, with a previous calibration, the integration into an online algorithm.

However, the derivation of a formula that supported the identification of the ideal threshold was an exploratory endeavor with the ultimate goal to facilitate threshold selection. The optimal thresholds for both tested algorithms were still selected manually upon inspection of the resulting values. We hope that both the results of experiment 1 and experiment 2 can contribute to the growing body of references for best-practices in gaze interaction in 3D Virtual Reality.

## Conclusion

Our study systematically compared the effects of both distance and number of objects in a smooth-pursuit selection task in Virtual Reality. Overall, performance was higher for the difference-based algorithm, suggesting that tasks relying on high reliability would benefit from the slightly higher time needed. The 3D difference-based algorithm also showed a higher robustness across all variations regarding object size and trajectory. Seeing that the influence of distance and therefore benefit of adding the third axis to the algorithms was mostly notable in very close proximity (0.4m). With close distances being advantageous for 3D algorithms, there is a trade-off between high detection rates user experience, as too many visible objects in close distances create discomfort for the user, as seen in the need for adaptations. Hence, we recommend to use closer distances if visible objects are limited in number, and further distances elsewise, as our experiments have shown that the decrease in detection performance seems to be stable for distances larger than 0.8m. As Khamis et al. (17) have shown, target size did not influence performance. We therefore recommend to keep targets as small as convenient to reduce the amount of visual flow in closer distances. However, while effects of larger distances than 1.4m should be neligible due to the parallelization of the eyes, further research to find the best possible range for depth tracking along the third axis is encouraged.

While our approach was based on a correlation-based and a difference-based algorithm, future research could further investigate the possible benefit of integrating the third axis into currently novel algorithms such as the slope method by Drewes et al. (8).

Furthermore we investigated the idea of an ideal threshold based on parameters of the environment. Future approaches could be refined to include additional factors either into design decisions or by adding to the threshold algorithm. Drewes et al. (10) demonstrated that optimal detection rates correspond to the individual user's target speed preference in 2D smooth pursuit tasks. The target speed in our study was selected based on the overall subjective preferences of users in a pre-study, but varied depending on object trajectory. Therefore, for interaction settings that require best possible detection rates, adapting to the users preferred speed might be beneficial.

While our study involved a constant task to be performed by the participants over all conditions, applications would have different levels of engagement and demands of the user. Kosch et al. (202018) have used the variation in deviations within gaze trajectories during smooth pursuit movements to successfully predict cognitive workload. Aside from using the workload information for adaptive experiences, the results of an online-classification could be used to inform the detection threshold as well, thus possibly further improving the algorithm performance.

## Acknowledgements

We acknowledge support by the German Research Foundation and the Open Access Publication Fund of TU Berlin. We wish to thank the Chair of Human Machine Systems at TU Berlin for providing the research facilities and technical equipment.

## supplementary material


