# Supplementary material for: A systematic performance comparison of two Smooth Pursuit detection algorithms in Virtual Reality depending on target number, distance, and movement patterns
Source: J Eye Mov Res. 2023 May 29;15(3):10.16910/jemr.15.3.9. doi: 10.16910/jemr.15.3.9 (PMC11321744; doi:10.16910/jemr.15.3.9)
Supplement: Supplementary file 1 [file jemr-15-03-i-SD1-01.pdf]

Supplementary Materials

Freytag, S.-C., Zechner, R. & Kamps, M. (2023). A systematic performance comparison of two Smooth Pursuit detection algorithms in Virtual Reality depending on target number, distance, and movement patterns. *Journal of Eye Movement Research, 15*(3):9. https://doi.org/10.16910/jemr.15.3.9

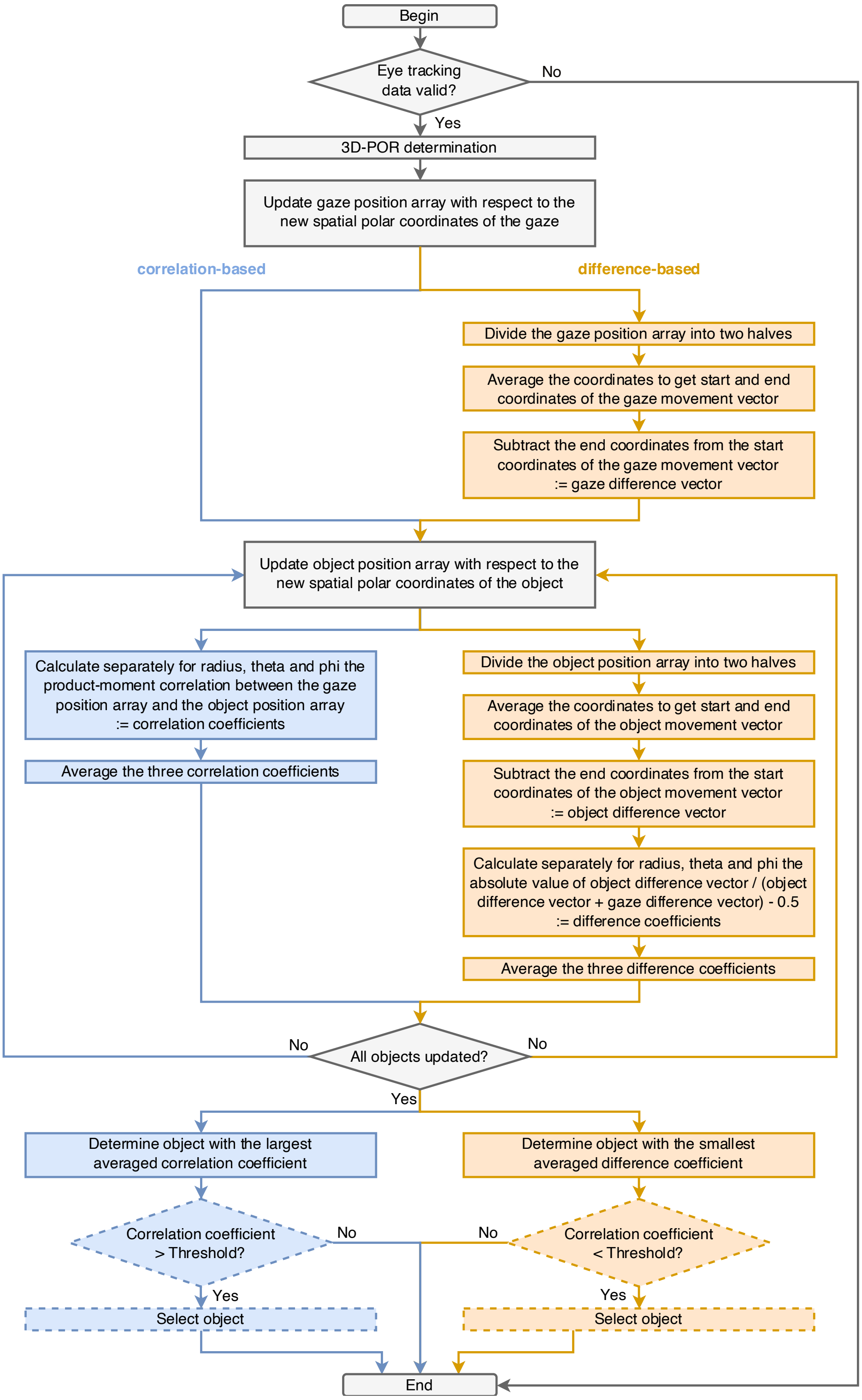

Figure S1. Workflow of the correlation-based and difference-based algorithms.

Table S1. Overview of optimal thresholds for each object configuration, based on DR, Efficiency_1 and Efficiency_2. Where no clear optimum value but tendencies were identifiable, values are presented in braces. Double values show thresholds of equal performance.

	threshold		threshold Efficiency1		threshold Efficiency2	
object config.	corr.	diff.	corr.	diff.	corr.	diff.
3A	.65	.13	.75	.13	.75	.08
3B	.65	.11	.75	.11	.65/.80	.08/.05
4A	.70	.14	.70	.14	.80	.05
4B	.80	.08	.80	.08	.80	.05
5A	.45	.11	.75	.10	.75	(.11)
5B	.70	.10	.80	.10	.80	.05
6	.70	.10	.85	.10	.70/.85	.09/.05
7	.75	.08	.85	.08	.85	.08
8A	.80	.08	.85	.07	.80	.06/.04
8B	.85	.09	.85	.07	.85	(.03)
9A	.75	.10	.85	.10	.75	.06
9B	.75	.08	.85	.08	.75	(.08)
10A	.80	.08	.85	.06	.80	(.03)
10B	.80	.08	.85	.07	.80	.08
11	.80	.09	.90	.06	.85	.04
12A	.80	.08	.90	.04	.85	.04
12B	.60	.08	.85	.06	.80	.08
M(all)	.75	.08	.85	.08	(.75)	(.08)
